# Purinergic P2X7 receptor mediates hyperoxia-induced injury in pulmonary microvascular endothelial cells via NLRP3-mediated pyroptotic pathway

**DOI:** 10.1515/med-2024-1097

**Published:** 2024-12-04

**Authors:** Wen Zeng, Zhuyu Deng, Huaying Li, Shuqiang Gao, Rong Ju

**Affiliations:** Department of Neonatology, Chengdu Women’s and Children’s Central Hospital, School of Medicine, University of Electronic Science and Technology of China, Chengdu, 611731, Sichuan, China

**Keywords:** hyperoxia, P2X7 receptor, NLRP3 inflammasome, pyroptosis, pulmonary microvascular endothelial cell

## Abstract

**Background:**

Hyperoxia-induced injury is a well-recognized cause of bronchopulmonary dysplasia (BPD). Existing research studies have not well elucidated the exact mechanisms underlying hyperoxia-induced cellular damage. This study examines the involvement of the P2X7 receptor (P2X7R) in hyperoxia-induced damage to human pulmonary microvascular endothelial cells (HPMVECs) via the NOD-like receptor family, pyrin domain-containing protein 3 (NLRP3) pathway.

**Methods:**

HPMVECs developing hyperoxia-induced injury were subjected to the treatment of either selective inhibitors or a P2X7R/NLRP3 agonist. Western blot analysis assisted in the quantification of the levels of P2X7R, NLRP3, caspase-1, and gasdermin D (GSDMD). Additionally, the release of TNF-α, IL-1β, and IL-18 was assessed by ELISA and qRT-PCR.

**Results:**

Exposure to hyperoxia diminished cell viability and escalated the levels of P2X7R, caspase-1, NLRP3, GSDMD, and N-terminal-GSDMD. This exposure notably increased the release of TNF-α, IL-1β, and IL-18 in HPMVECs. Notably, the suppression of P2X7R using the inhibitor A438079 decreased pyroptosis and inflammatory responses. Conversely, stimulation of P2X7R by 3′-*O*-(4-benzoylbenzoyl) adenosine 5′-triphosphate (BzATP) triggered pyroptosis, while inhibition of NLRP3 with glibenclamide ameliorated the damage induced by BzATP.

**Conclusions:**

The P2X7R/NLRP3 pathway crucially affects the hyperoxia-induced inflammation and pyroptosis in HPMVECs, hinting the potential of blocking P2X7R/NLRP3-mediated pyroptotic pathway as a valuable therapeutic strategy for BPD.

## 1 Introduction

Bronchopulmonary dysplasia (BPD) is a class of chronic lung disease characterized by high morbidity and mortality among preterm infants. It has been reported that 30–50% of extremely premature infants with birth weights below 1,000 g develop BPD [1,2]. Research indicates that injury to pulmonary microvascular structures disrupts both lung function and postnatal lung development, including angiogenesis and alveolarization, which are critical pathological features of BPD [3–5]. Yet, the specific pathological mechanisms of BPD remain elusive, and effective treatments are still lacking.

Pulmonary microvascular endothelial cells (PMVECs) constitute a primary component of the alveolar-capillary membrane and are crucial for vascular homeostasis, angiogenesis, and cellular communication [6]. Dysfunction in PMVECs can contribute to BPD development. Hyperoxia is recognized as a significant etiological factor in BPD. Studies have demonstrated that hyperoxia diminishes the bioactivity of PMVECs and hinders the formation of distal microvasculature [7,8]. Despite significant advances, a full understanding of the molecular dynamics through which hyperoxia inflicts damage in PMVECs remains elusive.

Hyperoxia is linked to a surge in inflammatory cytokines, critical in the etiology of BPD [9,10,11]. The NLRP3 inflammasome, encompassing NLRP3, the adaptor ASC, and caspase-1, serves as a key regulator of pathological inflammation [12,13]. Activation of NLRP3 instigates the pro-caspase-1 to be converted to its active form (caspase-1), facilitating the generation of IL-1β and IL-18, and initiating GSDMD-driven pyroptosis [13,14].

The purinergic receptor P2X7R, an adenosine triphosphate (ATP)-gated ion channel, is integral to multiple cellular processes (immune response, proliferation, and apoptosis). It is posited that P2X7R may trigger the NLRP3 inflammasome, precipitating caspase-1-mediated pyroptosis [15–17]. Found in diverse microvascular endothelial cells, including PMVECs [18,19], the activation state of P2X7R under hyperoxic conditions remains uncertain.

This research developed a hyperoxic model in PMVECs to delineate the function of P2X7R in hyperoxic injury and to evaluate the interplay between P2X7R and the NLRP3 inflammasome in modulating PMVEC pyroptosis.

## 2 Materials and methods

### 2.1 Cell culture and experimental design

HPMVECs (cat. no. BNCC360121; BeNa Culture Collection, China) were cultured following the supplier’s instructions. All things considered, the HPMVECs were cultured in a humidified 5% CO_2_ incubator at 37°C in endothelial cell medium (cat. no. 1001; ScienCell, San Diego, CA, USA) with 10% fetal bovine serum (cat. no. 25030081; Thermo Fisher Scientific, Waltham, MA, USA) and 100 U/mL penicillin/streptomycin (cat. no. C0222; Beyotime Institute of Biotechnology, Beijing, China). Cells were passaged every 2–3 days and used for passages 3–5.

There were five groups of HPMVECs randomly assigned to the study: control, hyperoxia, hyperoxia + A438079, BzATP, and BzATP + glibenclamide. Under normoxic circumstances (21% O_2_ and 5% CO_2_), the control, BzATP, and BzATP + glibenclamide groups were studied. Both the hyperoxia and hyperoxia + A438079 groups were exposed to a high level of oxygen (95% O_2_ and 5% CO_2_) for 24 and 48 h, respectively. Cells in the hyperoxia + A438079 group underwent 2 h of pre-incubation using 10 µM A438079 (cat. no. HY-15488; MedChemExpress, Monmouth Junction, NJ, USA) prior to hyperoxia; cells in the BzATP group received 200 µM BzATP (cat. no. HY-136254; MedChemExpress) for 2 h; and cells in the BzATP + glibenclamide group underwent 2 h of treatment using 200 µM BzATP and 50 µM glibenclamide (cat. no. HY-15206; MedChemExpress).

### 2.2 Immunocytochemistry (ICC)

To fix the HPMVECs, 4% paraformaldehyde was utilized, and to permeabilize them, 0.25% Triton X-100 was used. Cells received half an hour of inhibition using 1% bovine serum albumin (cat. no. 30063481; Thermo Fisher Scientific), followed by one night of pre-incubation using primary antibodies for CD34 (1:300; cat. no. sc-19621; Santa Cruz Biotechnology, Dallas, TX, USA) and Factor VIII (1:1,000; cat. no. ab275376; Abcam, Cambridge, MA, USA) at 4°C, and another 1 h of incubation using HRP-conjugated secondary antibodies (1:100, cat. no. A0208 and A0216; Beyotime Institute of Biotechnology). Diaminobenzidine (cat. no. sc-24982; Santa Cruz Biotechnology) was used to stain the cells for 5 min. Hematoxylin (cat. no. sc-24973; Santa Cruz Biotechnology) was then used as a counterstain. The photos were shot using an optical microscope (Leica, German).

### 2.3 Transmission electron microscopy (TEM)

Weibel-Palade bodies were detected by TEM. HPMVECs were subjected to 4 h of fixation in 2.5% glutaraldehyde in 0.1 M cacodylate buffer pH 7.4 at 4°C, followed by another 2 h of culture in medium added with 1% osmium tetroxide at 4°C. We used a graduated ethanol series of 30, 50, 70, 80, 95, and 100% to dehydrate the cells, and then embedded them in SPI-Pon^TM^ 812 epoxy resin (SPI Supplies, West Chester, PA, USA). Ultrathin sections of 70 nm thickness were prepared. Finally, a Hitachi HT7700 TEM (Japan) operating at 100 kV was used to observe certain ultrastructural features of sections stained using 3% uranyl acetate and 2.7% lead citrate.

### 2.4 Cell viability assay

The Cell Counting Kit-8 (CCK-8) assay (cat. no. CK04; Dojindo, Osaka, Japan) assisted in determining the cell viability as per the producer’s protocol. HPMVECs were seeded in 96-well plates (100 μL/well) to achieve 1 × 10^4^ cells per well, with each well added with 10 μL of CCK-8 reagents. Following 1 h of incubation, a microplate reader (Bio-Rad, Hercules, CA, USA) was adopted for measurement of the absorbance at 450 nm. The results were used to ascertain cell viability with a control value of 100%.

### 2.5 Quantitative real-time reverse transcription PCR (qRT-PCR) analysis

Following treatments, HPMVECs were treated with TRIzol Reagent (cat. no. 15596026; Thermo Fisher Scientific) for total RNA extraction, and cDNA was produced with the Takara Reverse Transcription Kit (cat. no. RR037A; Takara Bio, Kyoto, Japan). qRT-PCR was conducted using SYBR Green PCR Master Mix (cat. no. 1725150; Bio-Rad) (Table 1). Here we took GAPDH an internal control. Calculation of the relative expression relied on the 2^−ΔΔCq^ method.

**Table 1:** List of primers

Gene	Primer sequences	Product size (bp)
*GAPDH*	Forward: 5′-AGATCCCTCCAAAATCAAGTGG-3′	130
	Reversed: 5′-GGCAGAGATGATGACCCTTTT-3′	
*TNF-α*	Forward: 5′-ACTGAAAGCATGATCCGGGA-3′	141
	Reversed: 5′-GCAGAAGAGCGTGGTGGC-3′	
*IL-1β*	Forward: 5′-TCCGACCACCACTACAGCAAG-3′	91
	Reversed: 5′-GTGGGCAGGGAACCAGCATC-3′	
*IL-18*	Forward: 5′-AGATAGCCAGCCTAGAGGTATGGC-3′	119
	Reversed: 5′-TGATGTTATCAGGAGGATTCATTTC-3′	

TNF-α, tumor necrosis factor-α; IL-1β, interleukin-1β; IL-18, interleukin-18.

### 2.6 Enzyme-linked immunosorbent assay (ELISA)

ELISA kits were used to quantify the amounts of TNF-α, IL-1β, and IL-18 in the cultured HPMVECs’ supernatant (cat. no. ml077385, ml058059, and ml058055; Shanghai Enzyme-linked Biotechnology Co., Ltd, Shanghai, China). A microplate reader served for measuring the absorbance at 450 nm.

### 2.7 Western blot (WB) analysis

To conduct WB analysis, whole cell extracts were prepared. About 50 μg protein aliquot underwent 10% SDS-PAGE separation, and was moved onto a polyvinylidene difluoride membrane (Millipore, Billerica, MA, USA). Following 2 h blockage in 5% fat-free milk, blots received one night of culture using primary antibodies at 4°C. The following primary antibodies were used: anti-P2X7R (1:1,000; cat. no. 13809; Cell Signaling Technology, Danvers, MA, USA), anti-NLRP3 (1:1,000; cat. no. ab4207; Abcam), anti-Caspase 1(1:1,000; cat. no. 22915-1-AP; San Ying Biotechnology, Wuhan, China), anti-GSDMD (1:1,000; cat. no. ab210070; Abcam), anti-cleaved N-terminal-GSDMD (N-GSDMD) (1:1,000; cat. no. ab215203; Abcam), anti-GADPH (1:1,000; cat. no. ET1601-4; HuaAn Biotechnology Co., Ltd, Hangzhou, China), and anti-β-actin (1:1,000; cat. no. HA722023; HuaAn Biotechnology Co., Ltd). Following washing, the blots underwent another 2 h of incubation using secondary antibodies (1:1,000; Beyotime Institute of Biotechnology). The membranes were rinsed and targeted bands were identified using an enhanced chemiluminescence system (cat. no. 32109;Pierce, USA;). Labworks Analysis Software (Upland, CA, USA) was used for quantitative analysis of protein densitometry, which was then normalized to the levels of housekeeping proteins GADPH or β-actin.

### 2.8 Statistical analysis

Experimental data analysis relied on the SPSS 23.0, and data presentation followed mean ± standard deviation format. Statistical distinctions among groups were established through one-way ANOVA, succeeded by the least significant difference *t*-test. *P* < 0.05 reported statistical significance.

## 3 Results

### 3.1 Characterization of HPMVECs

**Figure 1:**
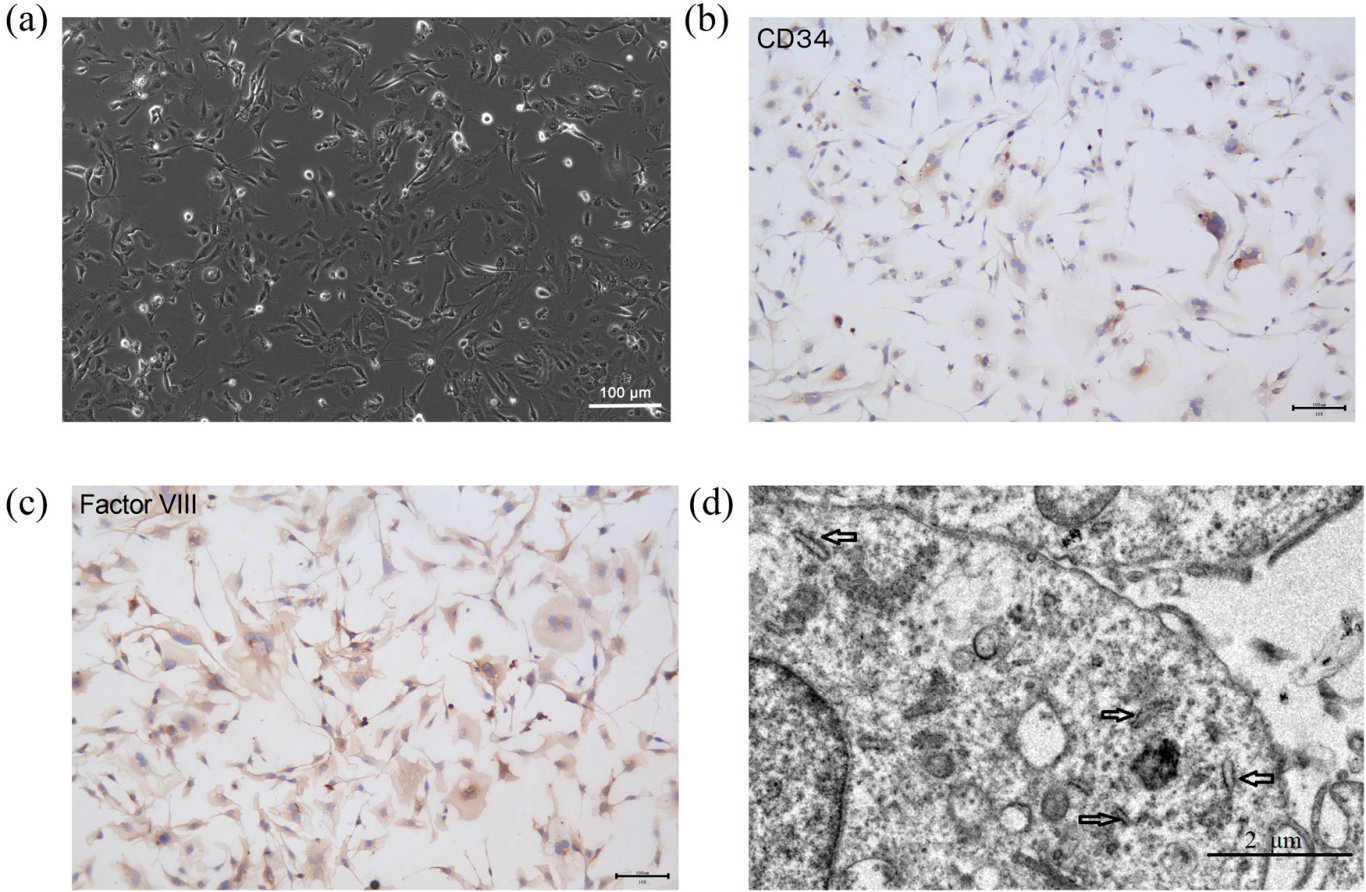
Culture and identification of HPMVECs. (a) HPMVEC morphology after 2 days in culture, observed under a light microscope. Scale bar = 100 μm. (b) and (c) ICC for endothelial cell-specific markers CD34 and Factor VIII. Both markers showed positive expression in cultured cells. Scale bar = 100 μm. (d) Ultrastructural morphology of Weibel-Palade bodies (indicated by arrows) in HPMVECs, observed by TEM. Scale bar = 2 μm.

Cultured HPMVECs were characterized by observing morphology, performing ICC, and using TEM. Phase-contrast light microscopy revealed that HPMVECs retained the classic cobblestone morphology characteristic of endothelial cells (Figure 1a). ICC staining confirmed the presence of endothelial-specific markers CD34 and factor VIII-related antigen in the cells (Figure 1b and c). Additionally, the morphology of Weibel-Palade bodies, specific morphological markers of endothelial cells, was observed by TEM. These rod-shaped organelles were present in cultured HPMVECs (Figure 1d).

### 3.2 P2X7R activation in hyperoxia-induced injury in HPMVECs

**Figure 2:**
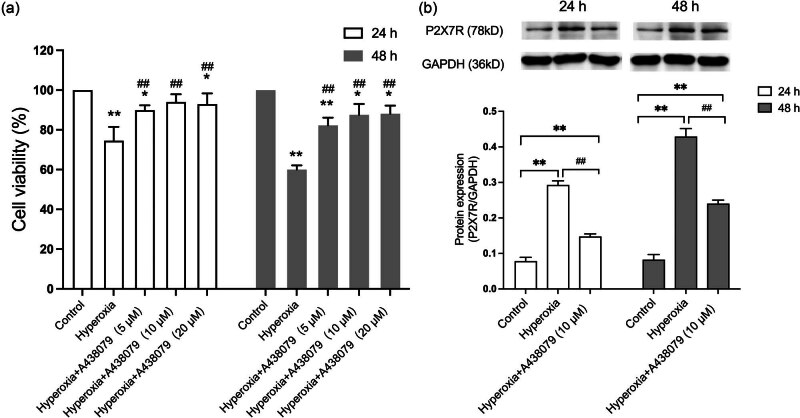
Effects of blocking P2X7R on HPMVECs in hyperoxia. (a) Cell viability assessed by CCK-8 assay. A438079, a P2X7R antagonist, was used at concentrations of 5, 10, and 20 μM. Viability was higher in the hyperoxia + A438079 groups than in the hyperoxia-only group. (b) Protein expression levels of P2X7R were determined by WB. GAPDH served as the loading control. Histograms represent the relative quantitative evaluation of protein levels. *P < 0.05 and **P < 0.01 versus the control group; ^#^ *P* < 0.05 and ^##^ *P* < 0.01 versus the hyperoxia group.

The impact of P2X7R on hyperoxic injury in HPMVECs was investigated by measuring cell viability with a CCK-8 assay. As depicted in Figure 2a, elevated exposure to hyperoxia was accompanied by obviously weakened cell viability (*P* < 0.01). To further explore the role of P2X7R during hyperoxia, the receptor was blocked with A438079, a P2X7R antagonist. It was found that cell death induced by hyperoxia was reduced by approximately 20 and 27% after 24 and 48 h, respectively, with 10 μM A438079 incubation, versus the hyperoxia group (*P* < 0.01). This concentration was used in subsequent analyses of P2X7R function.

P2X7R expression was then evaluated by WB analysis (Figure 2b). Hyperoxia was found to elevate the expression of P2X7R in HPMVECs (*P* < 0.01). Treatment with the inhibitor A438079 partially reversed the hyperoxia-induced elevation in P2X7R expression (*P* < 0.01), implicating that P2X7R participates in hyperoxic injury in HPMVECs.

### 3.3 Hyperoxia-induced pyroptosis in HPMVECs through P2X7R activation

**Figure 3:**
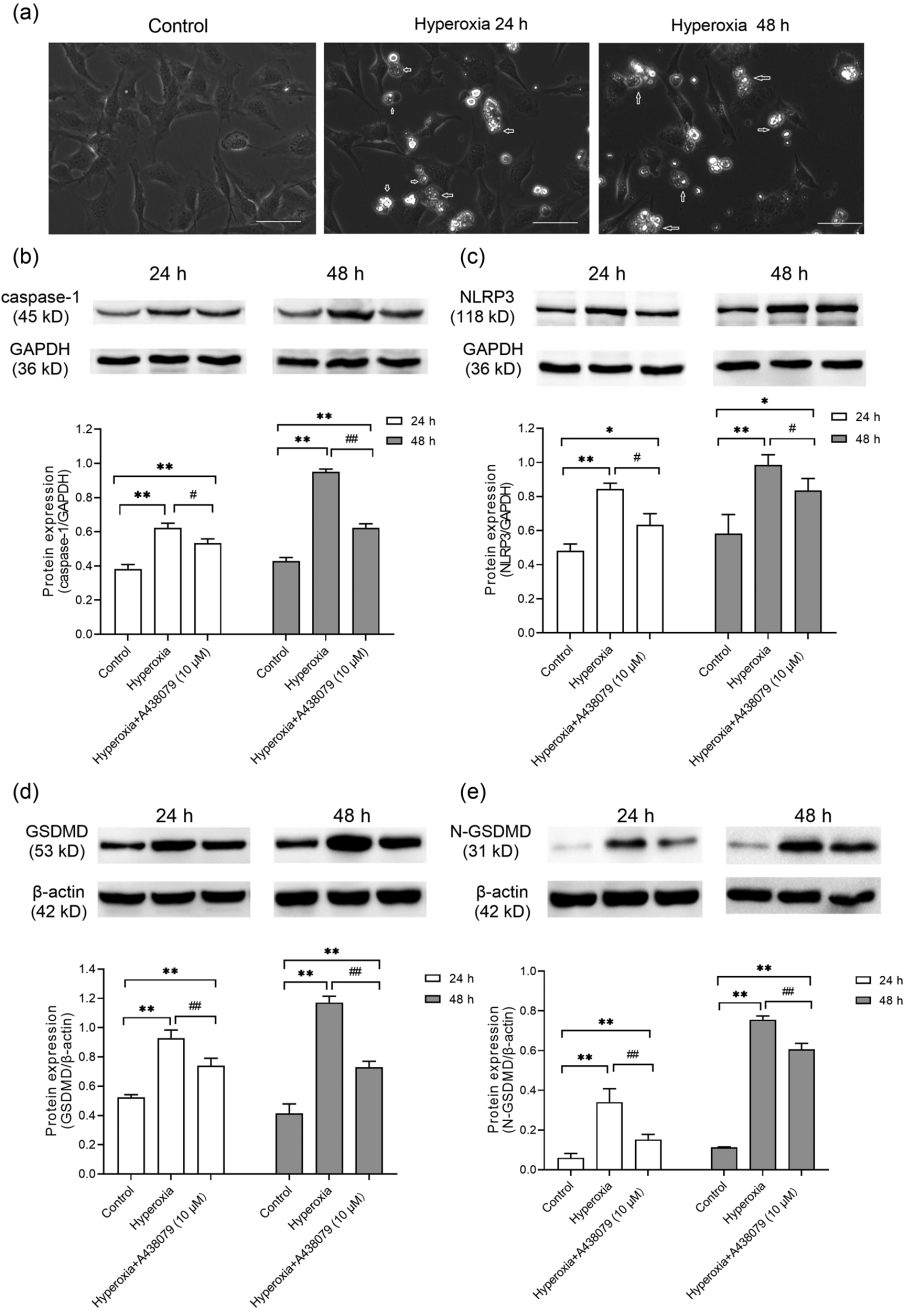
Effects of blocking P2X7R on NLRP3-mediated pyroptosis in hyperoxia. (a) Microscopic images of HPMVECs in control and hyperoxia groups, with arrows indicating cells exhibiting pyroptotic morphology. Scale bar = 20 μm. (b)–(e) Protein expression levels of caspase-1 (b), NLRP3 (c), GSDMD (d), and N-GSDMD (e) were determined by WB. GAPDH served as the loading control. Histograms represent the relative quantitative evaluation of protein levels. *P < 0.05 and **P < 0.01 versus the control group; ^#^ *P* < 0.05 and ^##^ *P* < 0.01 versus the hyperoxia group.

Pyroptosis was observed in the hyperoxia group (Figure 3a). The role of P2X7R in this process was then explored. P2X7R acts as a critical inflammation switch, mediating key downstream responses that activate inflammasomes, including the NLRP3 inflammasome. The NLRP3 inflammasome activates the protease caspase-1, inducing GSDMD-dependent pyroptosis.

WB analysis confirmed higher caspase-1, NLRP3, GSDMD, and N-terminal GSDMD levels in the hyperoxia group relative to controls (*P* < 0.01, Figure 3b–e). After administration of A438079, a decrease in the protein expression of these markers was noted, indicating that blocking P2X7R reduced NLRP3 inflammasome activation (*P* < 0.01, Figure 3b–e).

**Figure 4:**
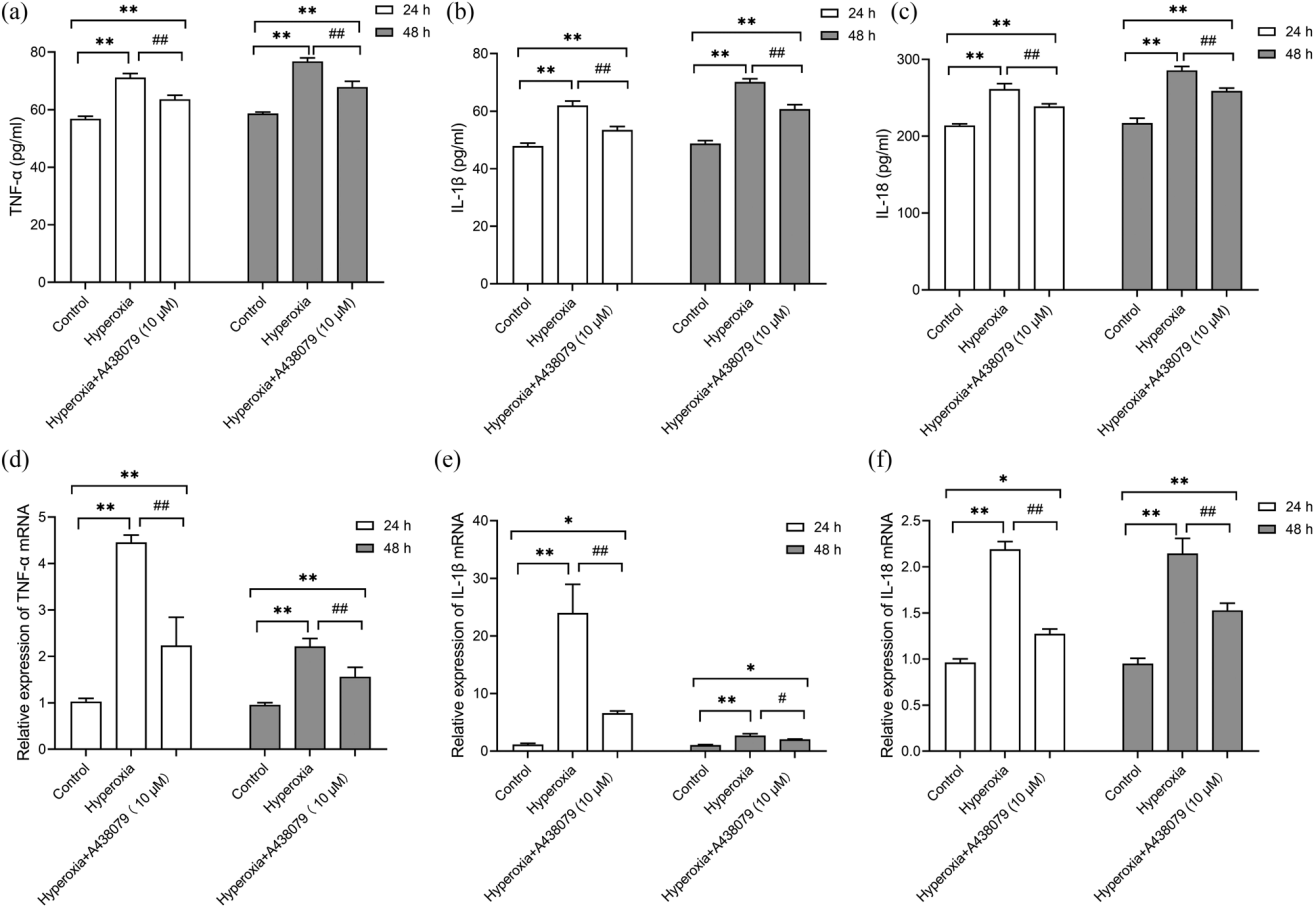
Effects of blocking P2X7R on proinflammatory factors in hyperoxia. (a)–(c) Protein levels of TNF-α, IL-1β, and IL-18, determined by ELISA. (d)–(f) Relative mRNA expression of TNF-α, IL-1β, and IL-18, assessed by RT-qPCR. *P < 0.05 and **P < 0.01 versus the control group; ^#^ *P* < 0.05 and ^##^ *P* < 0.01 versus the hyperoxia group.

Both ELISA and RT-PCR were employed to measure TNF-α, IL-1β, and IL-18 levels, aiming at exploring how pyroptosis affected the secretion of pro-inflammatory cytokines. According to analysis results, the hyperoxia group exhibited remarkably higher TNF-α concentrations versus the control at both mRNA and protein levels (*P* < 0.01, Figure 4a and d). Lower levels were observed following treatment with A438079 (*P* < 0.01). A similar pattern was noted for IL-1β and IL-18 (*P* < 0.01, Figure 4b, c, e, and f). Thus, these data suggest that P2X7R is associated with hyperoxia-induced pyroptosis and the downstream inflammatory response in HPMVECs.

### 3.4 Inhibition of NLRP3 alleviated HPMVEC injury induced by P2X7R activation

**Figure 5:**
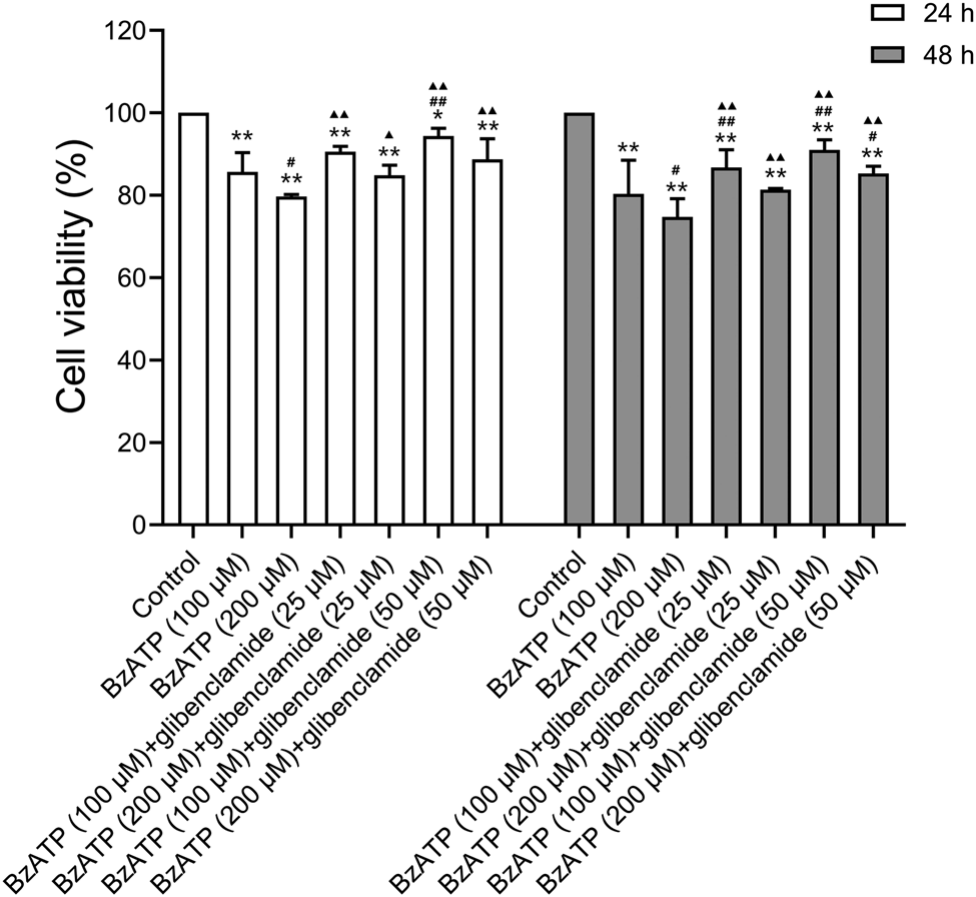
Effects of P2X7R/NLRP3 signaling on cell viability of HPMVECs. Cell viability assessed by CCK-8 assay. Cells were treated with BzATP, a P2X7R agonist, at concentrations of 100 and 200 μM. Glibenclamide, used to inhibit NLRP3 inflammasome activation at concentrations of 25 and 50 μM, ameliorated HPMVEC injury induced by P2X7R activation. *P < 0.05 and **P < 0.01 versus the control group; ^#^ *P* < 0.05 and ^##^ *P* < 0.01 versus the BzATP group (100 μM). ^▲^ *P* < 0.05 and ^▲▲^ *P* < 0.01 versus the BzATP group (200 μM).

The role of P2X7R was further verified by examining the cell viability of HPMVECs in response to the selective P2X7R agonist BzATP at concentrations of 25 and 50 µM. Dose–response effects showed decreased viability in the BzATP group (*P* < 0.01, Figure 5). The involvement of the NLRP3 pathway was tested using the NLRP3 antagonist glibenclamide at concentrations of 100 and 200 µM. Results demonstrated that glibenclamide alleviated BzATP-induced cell injury, supporting the NLRP3 pathway’s role downstream of P2X7R activation. Concentrations of 200 µM BzATP and 50 µM glibenclamide were selected for subsequent experiments.

### 3.5 P2X7R activated the NLRP3 inflammasome to induce pyroptosis in HPMVECs

**Figure 6:**
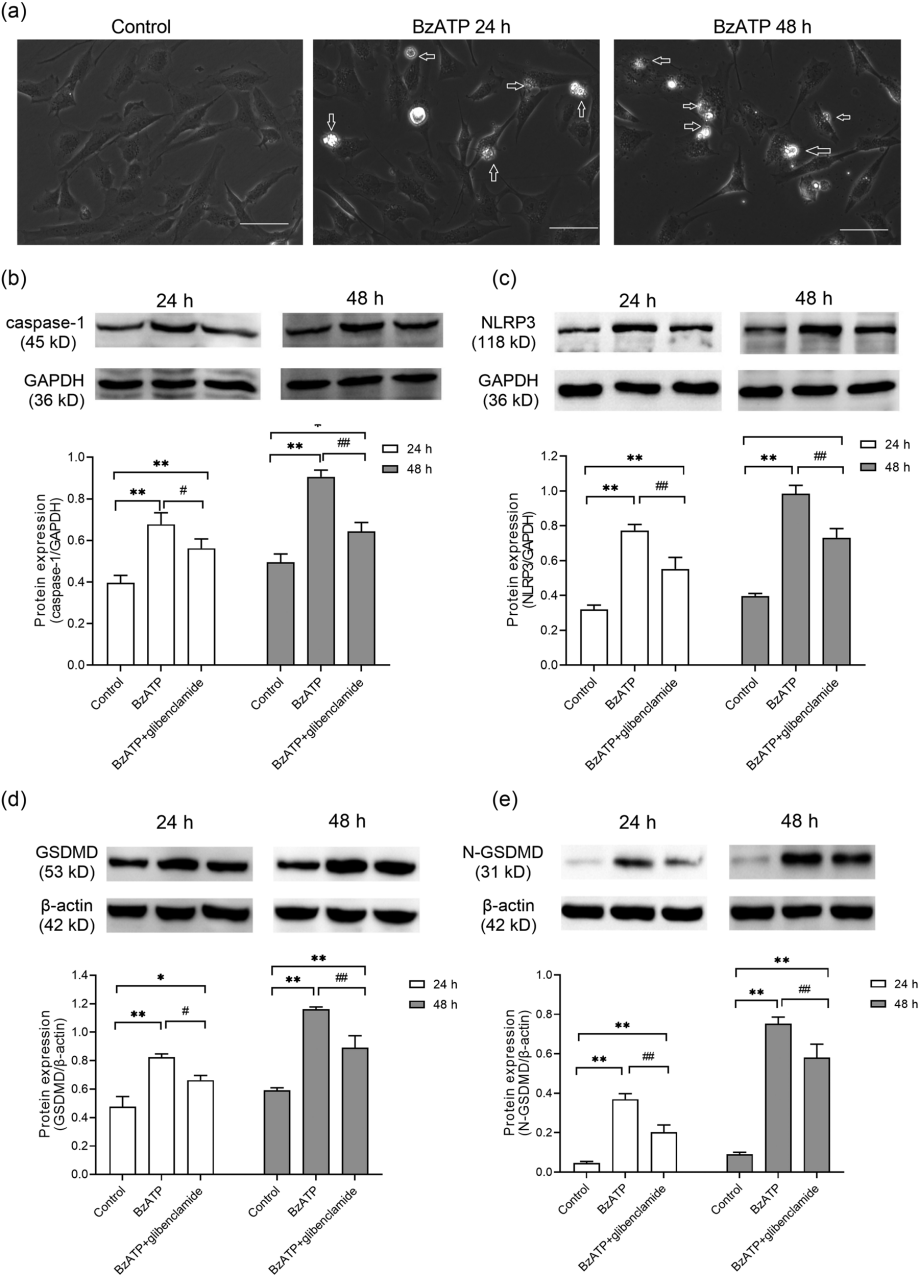
Effects of P2X7R/NLRP3 signaling on pyroptosis in HPMVECs. Cells were treated with BzATP (200 µM) to activate P2X7R. Glibenclamide (50 µM) was used to inhibit NLRP3 inflammasome activation. (a) Microscopic images of HPMVECs in control and BzATP treatment groups, with arrows indicating pyroptotic cells. Scale bar = 20 μM. (b)–(e) Protein expression levels of caspase-1 (b), NLRP3 (c), GSDMD (d), and N-GSDMD (e) were determined by WB. GAPDH and β-actin served as loading controls. *P < 0.05 and **P < 0.01 versus the control group; ^#^ *P* < 0.05 and ^##^ *P* < 0.01 versus the BzATP group.

**Figure 7:**
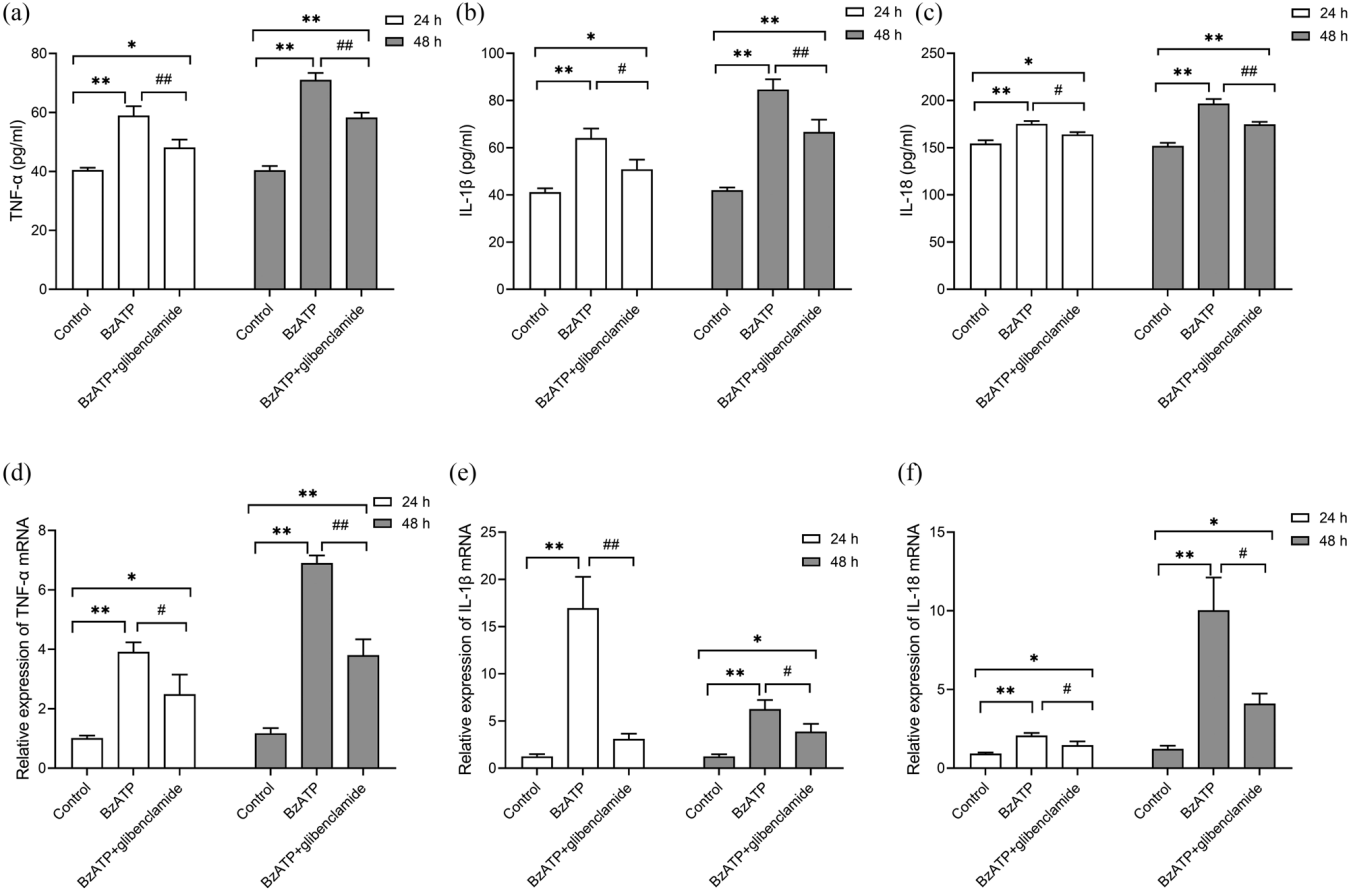
Effects of P2X7R/NLRP3 signaling on proinflammatory factors in HPMVECs. Cells were treated with BzATP (200 µM) to activate P2X7R. Glibenclamide (50 µM) was used to inhibit NLRP3 inflammasome activation. (a)–(c) Protein levels of TNF-α, IL-1β, and IL-18, determined by ELISA. (d)–(f) Relative mRNA expression of TNF-α, IL-1β, and IL-18, assessed by RT-qPCR. *P < 0.05 and **P < 0.01 versus the control group; ^#^ *P* < 0.05 and ^##^ *P* < 0.01 versus the BzATP group.

Finally, we investigated whether P2X7R activation affects HPMVEC viability through NLRP3 activation and pyroptosis. Stimulation of P2X7R with BzATP (200 µM) made pyroptosis-related markers more greatly expressed and promoted TNF-α, IL-1β, and IL-18 to be well secreted (*P* < 0.01, Figure 6). Conversely, the NLRP3 antagonist glibenclamide (50 µM) effectively reduced these expressions and secretions in HPMVECs (Figure 7). In summary, these results indicate that P2X7R may mediate pyroptosis through the NLRP3 inflammasome.

## 4 Discussion

This study probed the impact of P2X7R on hyperoxia-induced damage to HPMVECs *in vitro*. Findings indicate that hyperoxia notably decreases HPMVEC viability and elevates caspase-1, NLRP3, GSDMD, N-GSDMD, TNF-α, IL-1β, and IL-18 levels. Additionally, blocking P2X7R enhanced cell viability and reduced pyroptosis and inflammation following hyperoxia exposure. Moreover, treatment with glibenclamide to block NLRP3 significantly reduced pyroptosis induced by P2X7R activation. Our findings suggest a correlation between hyperoxic HPMVEC injury and the P2X7R/NLRP3-activated pyroptotic pathway.

Long-term exposure can disrupt normal pulmonary alveolarization and vascularization, increasing the risk of developing BPD. Previous research has indicated that impaired vascularization exacerbates alveolar simplification and contributes to pulmonary hypertension in BPD [20–22]. Therefore, strategies to protect PMVECs from hyperoxic injury may lead to effective therapeutic agents for BPD. In addition, it has been noted that infants with BPD have elevated levels of IL-1β, IL-6, IL-8, IL-10, IL-33, and TNF-α. This emphasizes the crucial role of inflammation in BPD progression [23–25].

P2X7R, a purinergic receptor, when stimulated, facilitates IL-1β and IL-18 to be released. In contrast, P2X7R-knockout mice do not release IL-1β following ATP stimulation [15]. P2X7R shows a strong expression in multiple cell types inside the lungs, including type I alveolar epithelial cells and PMVECs. In a mouse model of hyperoxic acute lung damage, its critical function in inflammatory processes has been confirmed [15,26]. Hyperoxia activates P2X7R, initiating K^+^ efflux and secretion of proinflammatory cytokines [27], suggesting its involvement in hyperoxic injury through the inflammatory response. However, research studies have not well explained its precise contribution to BPD progression. Our research endeavors to delineate the role of P2X7R in hyperoxic injury within PMVECs.

There is evidence to suggest that P2X7R strongly stimulates the NLRP3 inflammasome, resulting in inflammasome assembly and IL-1β and IL-18 release. The synthesis of IFN-γ, which is crucial for inflammatory responses and adaptive immunity, is facilitated by IL-1β, while IL-18 greatly promotes immune cell recruitment to damaged tissues [28]. Consequently, this research investigates whether P2X7R induces hyperoxic injury in HPMVECs via the NLRP3-mediated pyroptotic pathway. Pyroptosis, a caspase-dependent programmed cell death pathway, excels in producing pro-inflammatory factors (IL-1β and IL-18). Overactive pyroptosis can result in significant pathological harm. Numerous studies have underscored the association between pyroptosis and diverse inflammatory conditions, including sepsis, neurodegenerative disorders, inflammatory bowel disease, acute lung injury, non-alcoholic fatty liver disease, atherosclerosis, and cancer [29–34]. When pyroptosis occurs, the NLRP3 inflammasome sets off the canonical inflammasome pathway. The NLRP3 inflammasome, of which the activation could be achieved by exogenous or endogenous factors, as a result, caspase-1 became matured and IL-1β and IL-18 were secreted [12]. The release of N-GSDMD occurs upon the cleavage of GSDMD by activated caspase-1. After transferring to the plasma membrane, this N-GSDMD generates pores and triggers an inflammatory reaction. The conversion of caspase-1 precursors into corresponding active forms is facilitated by N-GSDMD, IL-1β, and IL-18 [35,36].

In this study, blockade of the P2X7R/NLRP3 pathway only partially alleviated hyperoxia-induced injury in HPMVECs, suggesting the involvement of other mechanisms. Various signaling pathways participate in pyroptosis. Gaidt et al. reported that Toll-like receptor (TLR) 4-TRIF-RIPK1-FADD-CASP8 signaling serves as an alternative pathway to activate NLRP3 inflammasome in human monocytes, independent of P2X7R activation [37]. He et al. showed that in murine dendritic cells, prolonged LPS exposure activated NLRP3 inflammasome and promoted IL-1β production via TLR signaling, in a P2X7-independent manner [38]. These findings indicate that NLRP3 is a convergent point for inflammatory cytokine release, some aspects of which may occur independently of P2X7R. Additionally, the NLRP1 inflammasome and NLRC4 inflammasome are also central in the canonical inflammasome pathway of pyroptosis [39,40]. Besides inflammasome-dependent pathways, other signaling mechanisms contribute to pyroptosis. Yersinia infections in macrophages trigger caspase-8 cleavage of GSDMD, which in turn initiates pyroptosis [41], and granzyme A, a lymphocyte-derived enzyme, cleaves GSDMB, which in turn triggers pyroptosis in a number of cancer cell types [42].

Furthermore, the pathways through which hyperoxia causes cellular damage are multifaceted and not entirely elucidated. Hyperoxia promotes endogenous reactive oxygen species (ROS) to be excessively produced, instigating an inflammatory response. Elevated ROS concentrations result in DNA damage, cell membrane lipid peroxidation, and the activation of genes associated with inflammation and cellular mortality [43]. In addition to pyroptosis, apoptosis represents another prevalent cell death mechanism triggered by hyperoxia. Various pathways, including Nrf2, NF-κB, and MAPK, can induce apoptosis upon exposure to hyperoxic conditions [44]. Thus, alleviating endothelial/epithelial cell apoptosis may well benefit the BPD treatment.

## 5 Conclusion

In summary, activation of P2X7R is implicated in hyperoxic injury of HPMVECs through NLRP3 inflammasome-related pyroptosis. These findings identify P2X7R as a potential therapeutic strategy targeting BPD prevention and treatment. Nevertheless, the P2X7R/NLRP3-mediated pyroptotic pathway is not the sole mechanism governing hyperoxic injury in HPMVECs, indicating the need for future studies to explore additional mechanisms involved.
